# Supplementary material for: An Effective Prophylactic and Therapeutic Protection Against Botulinum Type A Intoxication in Mice and Rabbits Using a Humanized Monoclonal Antibody
Source: Toxins (Basel). 2025 Mar 14;17(3):138. doi: 10.3390/toxins17030138 (PMC11946523; doi:10.3390/toxins17030138)
Supplement: Supplementary file 1 [file toxins-17-00138-s001.zip › toxins-3505915-supplementary.pdf]

Supplementary Materials: An Effective Prophylactic and Therapeutic Protection Against Botulinum Type A Intoxication in Mice and Rabbits Using a Humanized Monoclonal Antibody

Chi Ho Yu, Young-Jo Song, Dong Hyun Song, Hae Eun Joe, Chang-Hwan Kim, Hyungseok Yun, Na Young Kim, Euni Sim, Seong Tae Jeong and Gyeung Haeng Hur

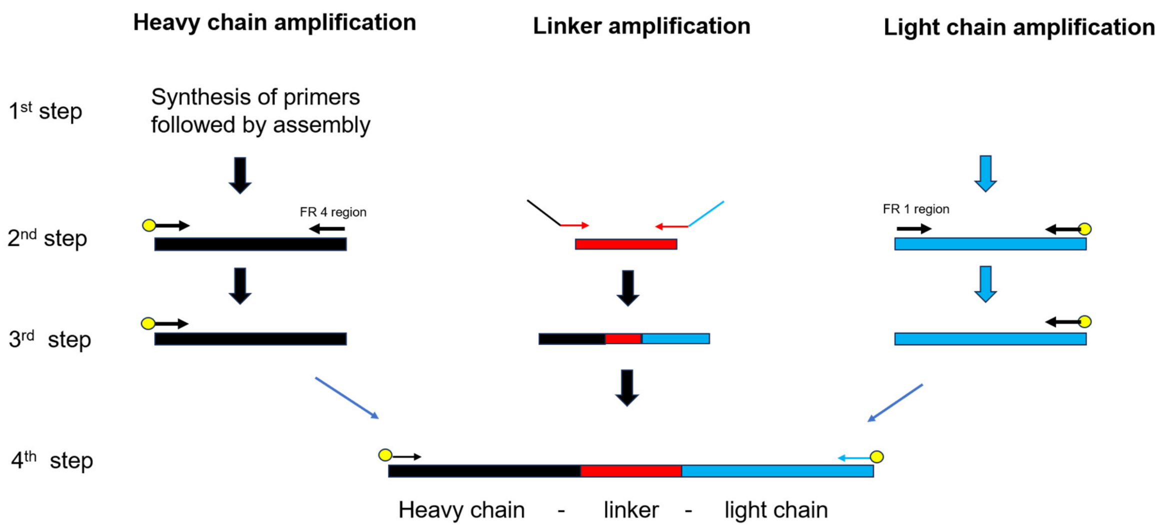

Figure S1. Schematic representation of overlapping PCR for scFv library construction. Sythesis and assembly were performed in step 1. Heavy linker and light chain wre amplified in step 2. Purification was performed in step 3. Overlapping and amplification were performed to get final PCR with restriction site.

 https://doi.org/10.3390/toxins17030138
